# The Use of Nitrate of Atropia for Producing Dilatation of the Pupil

**Published:** 1848-10

**Authors:** 


*The use of Nitrate of Atropia for producing Dilatation of the Pupil.*—Dr. Jacob remarks that, for application to the conjunctive, the solution of atropia, or some of its salts, is much more effective and convenient than any extract or tincture of belladonna. He has found that a single drop of a solution, made by dissolving two grains of nitrate of atropia in an ounce of water, dilated the pupil as perfectly, if not more perfectly, than the best extract of belladonna. It produced less pain and irritation, and was not attended with the inconvenience of leaving a string of green coagulum between the lids.
*Dublin Med. Press.*

